# Comprehensive Analysis of Geopolymer Materials: Properties, Environmental Impacts, and Applications

**DOI:** 10.3390/ma16237363

**Published:** 2023-11-27

**Authors:** Sami Sbahieh, Gordon McKay, Sami G. Al-Ghamdi

**Affiliations:** 1 Division of Sustainable Development, College of Science and Engineering, Hamad Bin Khalifa University, Doha P.O. Box 34110, Qatar; sasb41935@hbku.edu.qa (S.S.); gmckay@hbku.edu.qa (G.M.); 2 Environmental Science and Engineering Program, Biological and Environmental Science and Engineering Division, King Abdullah University of Science and Technology, Thuwal 23955, Saudi Arabia; 3 KAUST Climate and Livability Initiative, King Abdullah University of Science and Technology (KAUST), Thuwal 23955, Saudi Arabia

**Keywords:** life-cycle assessment (LCA), geopolymer concrete, compressive strength, durability, curing time, environmental impacts of geopolymers, applications of geopolymers

## Abstract

The advancement of eco-friendly technology in the construction sector has been improving rapidly in the last few years. As a result, multiple building materials were developed, enhanced, and proposed as replacements for some traditional materials. One notable example presents geopolymer as a substitute for ordinary Portland concrete (OPC). The manufacturing process of (OPC) generates CO_2_ emissions and a high energy demand, both of which contribute to ozone depletion and global warming. The implementation of geopolymer concrete (GPC) technology in the construction sector provides a path to more sustainable growth and a cleaner environment. This is due to geopolymer concrete’s ability to reduce environmental pollutants and reduce the construction industry’s carbon footprint. This is achieved through its unique composition, which typically involves industrial byproducts like fly ash or slag. These materials, rich in silicon and aluminum, react with alkaline solutions to form a binding gel, bypassing the need for the high-energy clinker production required in OPC. The use of such byproducts not only reduces CO_2_ emissions but also contributes to waste minimization. Additionally, geopolymer offers extra advantages compared to OPC, including improved mechanical strength, enhanced durability, and good stability in acidic and alkaline settings. Such properties make GPC particularly suitable for a range of construction environments, from industrial applications to infrastructure projects exposed to harsh conditions. This paper comprehensively reviews the different characteristics of geopolymers, which include their composition, compressive strength, durability, and curing methods. Furthermore, the environmental impacts related to the manufacturing of geopolymer materials were evaluated through the life-cycle assessment method. The result demonstrated that geopolymer concrete maintains positive environmental impacts due to the fact that it produces fewer carbon dioxide CO_2_ emissions compared to OPC concrete during its manufacturing; however, geopolymer concrete had some minor negative environmental impacts, including abiotic depletion, human toxicity, freshwater ecotoxicity, terrestrial ecotoxicity, and acidification. These are important considerations for ongoing research aimed at further improving the sustainability of geopolymer concrete. Moreover, it was determined that silicate content, curing temperature, and the proportion of alkaline solution to binder are the major factors significantly influencing the compressive strength of geopolymer concrete. The advancement of geopolymer technology represents not just a stride toward more sustainable construction practices but also paves the way for innovative approaches in the field of building materials.

## 1. Introduction and Background

**Figure 1:**
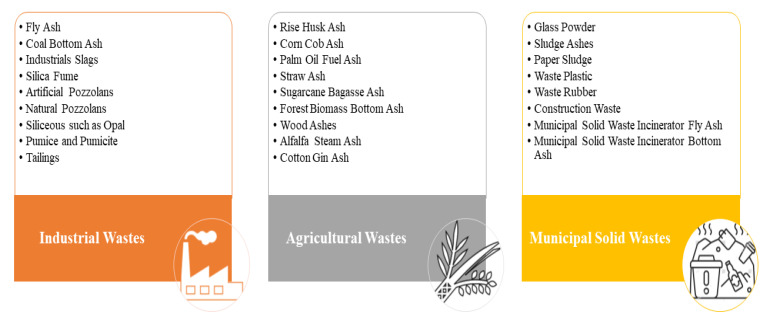
Solid wastes incorporated into geopolymers as potential aluminosilicate precursors.

Human activities and population growth raise the energy demands for construction materials and produce a substantial volume of solid waste across diverse sectors like steel and iron production, mining, power production, agriculture, and the production of electronic goods [1,2,3]. The handling and disposal of such waste streams have resulted in significant economic and environmental consequences. Hence, it is better to reuse or recycle some of the solid wastes into valuable resources, including construction materials, glass products, recycled energy, plastic products, and soil conditioners [4]. Over the past few years, there has been a substantial development in research focused on using solid waste in precursors, aggregates, fibers, and more [1]. Presently, alkali-activated materials (AAM), especially geopolymers (GPs), effectively make use of byproducts and industrial waste, often diverting them from improper disposal practices [5]. Therefore, geopolymers are emerging as a promising alternative for producing sustainable construction materials. This approach not only aids in waste management but also contributes to developing environmentally friendly construction solutions [6]. Ever since V. Glukhovsky’s initial discovery of alkali-activated binders in 1959 in Ukraine, extensive research has been dedicated to investigating and improving their physicochemical characteristics [7]. In the late 1970s, Davidovits introduced the expression “geopolymer” to characterize the inorganic polymeric system created through the metakaolin alkali activation [8]. Today, it stands as the most widely used term to refer to this material [9]. As per Davidovits [10], this innovative binder was produced through a modification of the techniques employed by the Romans and Egyptians. Davidovits goes as far as proposing that the pyramids might not have been constructed using natural stone but rather with man-made binders. His research suggests that the blocks of the pyramids were not composed of layers of calcium fossils, as is the case with natural stones. Instead, they were arranged randomly, much like in an artificial binder [10]. From 1979 to 1995, Davidovits and his team extensively contributed to the field of geopolymerization with numerous published papers and granted patents. Notably, they pioneered the creation of a silico-aluminate mineral polymer, which shaped as a solid solution at temperatures reaching approximately 120 °C [7]. Geopolymer is a groundbreaking aluminosilicate inorganic polymer characterized by an amorphous three-dimensional network structure consisting of silicon-oxygen and aluminum-oxygen tetrahedra interconnected through an oxygen bridge [11]. The material, resulting from the geopolymerization of an alkali activator and active aluminosilicate precursor, boasts several benefits, including exceptional mechanical strength, improved durability, resistance to both acid and thermal influences, cost-effectiveness, and better environmental impacts, including reduced CO_2_ emissions [12,13]. As depicted in Figure 1, the solid wastes incorporated into geopolymers as potential aluminosilicate precursors can be primarily categorized into three main groups: agricultural wastes (AW), municipal solid wastes (MSW), and industrial wastes (IW) [14]. Solid wastes containing aluminosilicate are often referred to as supplementary cementitious materials (SCMs). These materials have the potential to replace cement while maintaining similar effectiveness [15]. Yet, measuring the performance of SCMs directly is a complex task. This complexity arises from the challenge of identifying how, or in which combination, these SCMs modify the properties of the cementitious materials, a process that indirectly reflects their Degree of Reaction (DOR) [16]. The DOR of SCMs within hydrated Portland cement plays a significant role in formulating concrete with reduced carbon dioxide content. Numerous research efforts have been dedicated to finding ways to predict the DOR of SCMs in such a hydrated environment. A notable contribution in this field is by Degefa and colleagues, who developed a predictive model using a machine learning algorithm. This model, based on genetic programming and tailored for physical systems’ identification, paves the way for creating more environmentally sustainable and effective concrete designs, in line with current ecological goals [16]. Furthermore, explorations into thermodynamic modeling effectiveness have been conducted to predict the DOR of SCMs in hydrant Portland cement. These studies have revealed that this approach can yield fairly accurate predictions regarding bound water content and the DOR of SCMs [17]. Additionally, it has been observed that the reactivity of supplementary cementitious materials (SCMs) in cement mixtures remains active over an extended period. This ongoing reactivity plays a crucial role in enhancing the strength of the blend during prolonged hydration processes [18]. Such observations underscore the need to factor in elements like the water-to-cement ratio, the duration of curing, the composition of oxides, and the use of thermodynamic modeling for accurately predicting the DOR of SCMs in hydrated Portland cement.

Implementing GP technology in the building industry provides the potential for improved environmental impact at the construction material level [19]. Earlier studies have demonstrated that geopolymer concrete (GPC) surpasses conventional concrete (CC) in terms of environmental impact when employed as an alternative material [20]. Coal fly ash (CFA)-based geopolymers are increasingly recognized as an eco-friendly substitute for conventional Portland cement in concrete [21]. The process of geopolymerization presents an opportunity to replace cement with coal fly ash in construction, aiding in the pursuit of sustainable development [22]. Incorporating coal fly ash into concrete significantly affects how water moves within the concrete’s structure. Research indicates that substituting cement binders with CFA reduces water absorption levels, especially when the replacement is up to 35% [23]. Nevertheless, the impact of CFA on concrete’s characteristics is contingent on the proportion of CFA incorporated. Furthermore, Diatomaceous earth presents potential in the development of geopolymer concrete. It is characterized by its abundant silica content derived from fossilized algae [24]. The reviewed studies suggest that incorporating diatomaceous earth can lead to the creation of eco-friendly, insulating, and lightweight construction materials, thereby reducing the detrimental environmental and economic impacts associated with industrial solid waste [25]. Additionally, the research by Kipsanai et al. [26] delved into the properties of geopolymer concrete made with alkaline-activated diatomaceous earth and reinforced with sisal fibers. The outcomes reveal that sisal-reinforced geopolymer concrete possesses impressive mechanical, physical, and durable characteristics, underscoring its viability as an eco-friendly and resilient material for construction.

Moreover, optimum particle packing in concrete has been a focal point in recent research, particularly for its role in enhancing the macro and micro properties of sustainable concrete and geopolymer concrete. This approach, which involves analytical models for particle packing, has been instrumental in optimizing concrete mix designs. The benefits include a substantial reduction in cement usage, which can be cut down by over 50%, and a corresponding decrease in CO_2_ emissions by approximately 25% [27]. This strategy not only curtails environmental impact but also enhances workability and strength [27]. A notable study focused on the potential of creating eco-friendly concrete using materials readily available in the United Arab Emirates market. This research delved into the impact of optimal particle packing on both macro and micro aspects of sustainable concrete [28]. It incorporated the EMMA method for particle packing and conducted a comprehensive analysis encompassing mechanical properties, chemical composition, and cost considerations. The findings from the study underscored the significant potential of particle packing optimization in crafting sustainable, high-strength concrete formulations [28]. In another research by [29], the mechanical and durability characteristics of both traditional and high-strength geopolymer concrete were examined, employing the principles of particle packing theory, revealing that this method notably enhances the concrete’s compressive strength, durability, and workability.

The life-cycle assessment (LCA), an analytical tool used for evaluating the environmental impacts of products or services throughout their entire lifespan [30,31,32,33], has been employed to analyze the environmental aspects of GPC [34,35,36]. Within the life cycle of GPC, the primary environmental challenges have been linked to the utilization of alkali activators [34,35,37].

For the purpose of achieving a GP with optimal compressive strength (CS), several crucial factors need to be considered during the design phase. These factors include the selection of the aluminosilicate source, its specific composition, as well as the alkaline activator concentration and composition [38,39]. Additionally, determining the appropriate water content and deciding whether to cure at ambient or high temperature are equally significant variables in the process [40,41]. It is important to carefully address these aspects to successfully formulate a high-strength geopolymer [42]. The escalating attention towards geopolymers echoes the worldwide momentum toward sustainable development. This trend is particularly found in the construction industry, which is on a quest for greener substitutes to traditional building materials [43]. Geopolymers are becoming increasingly popular, not merely as an innovative category of materials but also as a symbol of the movement toward a more sustainable and resource-efficient tomorrow [44].

In this research, several key gaps in the field of sustainable construction materials, specifically geopolymer concrete (GPC), are addressed. Firstly, it undertakes a comprehensive environmental impact analysis of GPC using the life-cycle assessment (LCA) method, going beyond carbon footprint assessment to include minor impacts like abiotic depletion and ecotoxicity. Secondly, the study delves into the technical advantages of GPC over ordinary Portland concrete (OPC), focusing on aspects like enhanced mechanical strength, durability, and stability under diverse environmental conditions. Additionally, it explores the crucial factors influencing the compressive strength of GPC, such as silicate content, curing temperature, and the alkaline solution-to-binder ratio, areas not fully understood previously. The research also highlights the need for standardizing GPC production methods and a deeper understanding of its long-term durability. Addressing these gaps not only contributes to the advancement of geopolymer technology but also paves the way for more sustainable and innovative practices in the construction industry.

**Figure 2:**
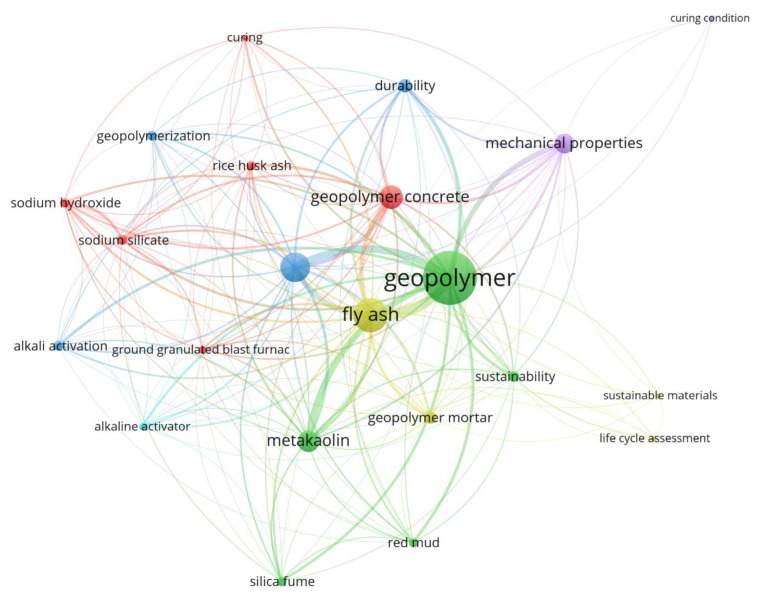
Bibliometric network visualization.

Figure 2 presents the results of the bibliometric network visualization, which offers a visual representation of the research focuses and interconnections within the field of geopolymers. In this visualization, each item is depicted by its label and typically represented by a circle. The size of both the circle and the label was decided by the weight assigned to the respective item. The network visualization was generated using the VOS viewer version 1.6.19, a specialized software for analyzing document measurement networks [45]. It was applied to analyze the terminology used in the titles of research articles related to geopolymers indexed by the Scopus database. 

### Aim and Research Significance

The primary aim of this research is to conduct a comprehensive review of geopolymer concrete (GPC), focusing on its composition, mechanical properties, and environmental impact. The study seeks to evaluate the sustainability of GPC as a substitute for ordinary Portland concrete (OPC), analyzing its potential to reduce CO_2_ emissions and other environmental pollutants in the construction industry. Key aspects such as the compressive strength, durability, and various factors influencing these properties, like silicate content and curing conditions, are explored in depth. Additionally, the research aims to assess the environmental impacts of geopolymer production using the life-cycle assessment (LCA) method, identifying areas where GPC excels and areas where it may have unintended negative impacts.

The significance of this research lies in its contribution to the field of sustainable construction materials. By providing an in-depth review of geopolymer concrete, the study highlights its potential as a more environmentally friendly alternative to traditional OPC, emphasizing its lower carbon footprint and ability to utilize industrial byproducts. The findings of this research are particularly crucial in informing the construction industry of the technical and environmental advantages of GPC, aiding in the transition toward more sustainable building practices. Furthermore, the identification of minor negative environmental impacts of GPC, such as abiotic depletion and ecotoxicity, underlines the need for ongoing research to optimize its composition and production processes. This study also addresses the current challenges and future research directions in geopolymer technology, thereby guiding future investigations and technological advancements in this field. Ultimately, the research underscores the importance of developing sustainable building materials like GPC in mitigating the environmental impact of the construction industry and contributing to global efforts against climate change. This paper is anticipated to serve as a beneficial resource for professionals, engineers, and researchers in the field of construction materials.

## 2. Geopolymers Composition

A geopolymer is formed by connecting AlO_4_^−^ and SiO_4_^−^ tetrahedra, wherein each tetrahedron shares its corners with another tetrahedron through oxygen atoms, creating a 3D structure. This resulting structure is primarily amorphous, though it might contain a few zeolitic phases. The amorphous portion is referred to as N–A–S–H gel, named after the ultimate composition of the geopolymerization output (Na_2_O−Al_2_O_3_−SiO_2_−H_2_O). Geopolymers are inorganic materials with polymeric structures produced by blending an alkaline solution with a dry solid, typically an aluminosilicate rich in Al and Si [46].

For geopolymer solidification, the presence of aluminum (Al) is crucial. Mixtures containing elevated levels of alkali silicate concentration tend to be metastable since the silica tetrahedra are susceptible to water-attacks. This leads to the creation of silanol (Si–OH) pairs (Equation (1), leading eventually to the creation of Si(OH)_4_ (Equation (2)). However, in an alkaline environment, Equation (2) dominates, causing the remaining oxygen bonds to weaken and the continued dissolution of the silicate. Hence, soluble silica alone is not sufficient for chemical hardening [47].

(1) $$-O-\begin{array}{c} \begin{array}{c} |\\ O\\ | \end{array}\\ Si\\ \begin{array}{c} |\\ O\\ | \end{array} \end{array}-O-\begin{array}{c} \begin{array}{c} |\\ O\\ | \end{array}\\ Si\\ \begin{array}{c} |\\ O\\ | \end{array} \end{array}-O-+H_{2}O⇌-O-\begin{array}{c} \begin{array}{c} |\\ O\\ | \end{array}\\ Si\\ \begin{array}{c} |\\ O\\ | \end{array} \end{array}-OH\;HO-\begin{array}{c} \begin{array}{c} |\\ O\\ | \end{array}\\ Si\\ \begin{array}{c} |\\ O\\ | \end{array} \end{array}-O-$$

(2) $$Si{\left(OH\right)}_{4}+{OH}^{-}⇌Si\left(OH_{3}\right)O^{-}+H_{2}O$$

The process of geopolymerization involves forming silicon–oxygen–silicon and silicon–oxygen–aluminum bonds owing to the links of aluminate and silicate tetrahedra. However, no aluminum–oxygen–aluminum bonds form due to their energetic instability. This indicates that the ratio of Si/Al could have a minimum value of 1. Sufficient aluminum content is essential to prevent silica dissolution [47].

The activating solution alkalinity leads to dissoluting aluminosilicates in the source material. In the process of molecular organization, some Si tetrahedra could be substituted with Al tetrahedra, resulting in negatively charged Al tetrahedra. These charges are balanced by positively charged alkaline cations from the activating solution [48]. In the N–A–S–H gel, silicon primarily plays a role in the formation of zeolitic nuclei. It plays a crucial role in the early stages, especially with water glass (SS) as the activator, undergoing initial dissolution to supply monomers for silica-rich gel formation. However, excessive dimers in the silicate can lead to faster yet more metastable gel formation [49]. Aluminum is actively involved in initial chemical reactions. A source rich in alumina releases more Al into the solution, enhancing source reactivity. An excess of Al can lead to reactions with crystalline products. Dissolved Al becomes part of the Si-rich gel structure, increasing its stability [49]. Sodium acts as a charge balancer, stabilizing the gel by balancing Al monomers or filling pores in mixtures with zeolitic products [49]. Al species transform to Al(iv) (or Al(OH)_4_^−^) during source material activation, providing an indication of unreacted aluminosilicate. Crystalline phases, often zeolites, may form under specific conditions, increasing with higher alkalinity and lower soluble silica content. A high water content aids full hydration and minimizes interactions between ion pairs, allowing for unimpeded growth of gel precipitates [50]. In summary, geopolymer formation depends on the interplay of Al and Si, with sodium balancing charges. The Si/Al ratio, aluminum content, and alkalinity affect the process, determining the characteristics of the resulting material. Additionally, factors like temperature and reaction time can lead to the formation of crystalline phases within the geopolymer structure.

## 3. Preparation of Geopolymer Concrete

**Figure 3:**
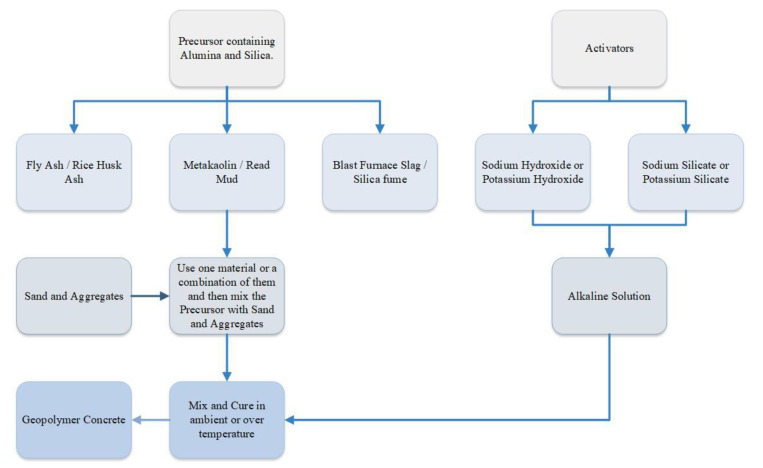
Flowchart for the process of creating a geopolymer cement concrete.

Geopolymers are inorganic polymer materials created through the combination of various aluminosilicate source materials with alkali-activator solutions [51]. Geopolymer cement concrete (GPCC) is a form of concrete produced with GP binder rather than ordinary Portland cement (OPC) [34]. The main components of GPC are source materials, alkaline activators mixed with fine or coarse aggregates, and water [52]. The aluminosilicate source materials may occur naturally, like metakaolin (MK), kaolin, bagasse ash, volcanic rock powder, and rice husk ash (RHA), or they might be produced industrially, like blast furnace slag (BFS), fly ash (FA), and silica fume (SF) [53]. The primary alkaline activators (AAs) are sodium hydroxide (NaOH) and sodium silicate (Na_2_SiO_3_); nevertheless, any silicate or hydroxide can be employed as an activator, including potassium silicate (K_2_SiO_3_) and potassium hydroxide (KOH) [52,54]. GPCC might be one-part or two-part based on the activator source addition process. The one-part GPC, commonly referred to as “Just Add Water,” requires solely a dry mix along with water. This dry mix is made by combining a solid alkali activator accompanied by a solid aluminosilicate precursor, with the option of including or excluding the calcination process [55]. Additionally, in the two-part GPCC, also known as conventional GPCC, the activators are introduced as a liquid state along with the water into a solid aluminosilicate precursor [54]. Figure 3 shows the flowchart for the process of creating a geopolymer concrete (GPC).

### 3.1. Aluminosilicate Precursors 

GPCC uses aluminosilicates, which are industrial and natural byproducts containing amorphous silica and alumina. In underdeveloped nations, materials from industrial and agricultural waste, including amorphous silica and alumina, are frequently utilized for energy production. Repurposing these wastes in the manufacturing process of geopolymer technology and cement-based products offers a potential solution to the issue of ash disposal [56]. 

#### 3.1.1. Fly Ash (FA)

Coal fly ash is a commonly available anthropogenic material produced in thermal power plants as a byproduct of coal combustion [57]. This industrial waste can create various environmental problems if released into the environment [58]. FA composition varies greatly based on the burnt coal type, combustion process, conditions, and cooling control [59]. However, it typically contains amounts of iron oxide (Fe_2_O_3_), calcium oxide (CaO), silicon dioxide (SiO_2_), aluminum oxide (Al_2_O_3_), and other minor components [60]. As per the guidelines presented by the American Society for Testing and Materials (ASTM) [61], FA is divided into two primary types: C and F fly ash. Class F is originated from the combustion of either anthracite or bituminous coal. It has a pozzolanic nature, with CaO content of under 18%, while class C has pozzolanic and self-cementing properties, produced from sub-bituminous coal or the burning of lignite, and has more than 18% of CaO [62]. Fly ash has several beneficial applications in multiple areas of the construction industry, such as the stabilization of soil, brick and block manufacturing, cement substitution in concrete, structural fill and embankment, constructing roads, asphalt pavement, and in dams [63]. There are several trace elements found in coal that are extremely toxic to both people and other living things. The obtained fly ash after combusting the coal has higher concentrations of these elements; therefore, fly ash is thought to have a negative environmental impact if not appropriately managed [64]. The correct management and utilization of fly ash may provide economic and environmental advantages while limiting harmful effects on the environment.

#### 3.1.2. Blast Furnace Slag (BFS)

The production of iron in blast furnaces results in the generation of blast furnace slag (BFS) as a byproduct. Iron ore, coke, and limestone are used to feed the furnaces. During the procedure, iron ore is converted into iron, and the remaining components combine to produce the slag. The created slag is then extracted as a molten liquid and allowed to cool [65]. There are two primary forms of BFS: granulated blast furnace slag (GBFS) and air-cooled blast furnace slag (ABFS). GBFS is made by quickly cooling molten slag with water or steam, which creates a glassy, granular material. ABFS is formed by enabling molten slag to cool slowly in the open air, which leads to a denser, more crystalline substance [66,67]. BFS is used as a substitute for cement, reducing the amount of clinker required for cement production. BFS cannot substitute cement completely; however, partial cement replacement gives good results and a greener approach in the construction field [68]. Additionally, BFS can serve as a suitable material for the production of geopolymer due to its high alumina and silica content [69], which might be considered a solution for industrial waste and a promising approach for developing sustainable materials.

#### 3.1.3. Silica Fume (SF)

Silica fume (SF), which is additionally called microsilica, is a valuable byproduct derived by an electric-arc furnace (EAF) throughout the manufacturing of silicon (Si) and ferrosilicon (FeSi) alloys [70]. SF comprises of very small particles, each with an average diameter of 0.1 µm [71]. This extremely small size of silica fume particles enables them to fill the voids that would otherwise remain unfilled. This characteristic results in a denser microstructure, contributing to elevated strengths, enhanced durability, and reduced permeability in materials [70]. Moreover, SF is a highly reactive pozzolan due to its chemical, mineralogical, and physical properties, which may be derived from natural or artificial sources. Additionally, whether with low or high silica content, SF possesses a nano-porous formation, serving as a valuable supplementary substance for GP within concrete applications [71]. The study carried out by Okoye et al. revealed that introducing silica fume produces an improvement in the compressive strength of the GPC produced. Additionally, increased flexural and tensile strengths were observed with escalating levels of SF content [72]. Hence, adopting silica fume fulfills a crucial role in enhancing the characteristics of the geopolymers, paving the way for more sustainable and innovative alternatives to traditional cement-based materials.

#### 3.1.4. Metakaolin (MK)

Metakaolin (MK) is an essential material used for producing geopolymers. It is mainly a pozzolanic material created from kaolin (China clay) clay through calcination at high temperatures (600 to 900 °C), where it undergoes amorphization and develops into a material with high reactivity [73]. Examinations of durability indicate that metakaolin geopolymers have enhanced characteristics with regard to water resistance, thermal resistance, and resistance against corrosion. [74]. The metakaolin-based geopolymers displayed enhanced workability in comparison to OPC as the proportion of sodium silicate (Na_2_SiO_3_) to sodium hydroxide (NaOH) was elevated up to a particular level. Beyond that level, workability decreased due to the elevated mixture viscosity [75,76]. The MK-based geopolymers’ mechanical characteristics were examined using orthogonal tests by Dai et al. The findings revealed that sodium- and potassium-based geopolymers displayed enhanced compressive and flexural strength compared to other binding materials [77]. Metakaolin serves as a primary product; thereby, its production remains unaffected by market fluctuations in other sectors. Nonetheless, despite its global availability, the current metakaolin production volume remains insufficient to adequately satisfy the global need for pozzolanic materials in the production of cement and concrete [70]. 

#### 3.1.5. Rice Husk Ash (RHA)

The rice husk ash (RHA) is a byproduct obtained by cultivating and processing rice. About 20–25% of the rice paddy comprises of an outer husk that cannot be digested. This husk is often separated and burned in nearby power facilities to provide steam for parboiling rice, in domestic stoves, or as fuel for producing electricity. Burning these husks turns them into ash, constituting roughly 18% of their original weight. Consequently, the production of one ton of rice yields around 45 kg (70 lb) of rice husk ash [70,78,79]. This ash contains a high silica content (between 80% and 95%) and distinctive pozzolanic properties [80]. Concrete containing rice husk ash (RHA) could extend the setting time of the cementitious paste while also improving the workability of the concrete mixture in comparison to OPC. Additionally, GPC made with RHA has the capacity to minimize the permeability and overall porosity of the concrete [71]. The utilization of RHA in geopolymers has gained remarkable interest recently due to its potential to enhance the mechanical characteristics, sustainability, durability, and cut some of the production costs compared to OPC [81].

#### 3.1.6. Red Mud (RM)

Red mud (RM) is a byproduct material produced during the extraction of alumina from bauxite via Bayer’s process [82]. Between roughly 1 and 2.5 tons of RM are generated for every 1 ton of alumina extracted, accounting for approximately 55% to 65% of the processed bauxite [83]. RM possesses distinctive properties, including a high pH level ranging from 10 to 12.5 [52], a substantial solids content spanning between 15% and 30%, and a varying chemical composition. Notably, its red color arises from its iron oxide content (Fe_2_O_3_), which varies between 20% and 60%. The additional components include aluminum oxide (AI_2_O_3_) at 10–30%, silicon dioxide (SiO_2_) at 2–20%, sodium oxide (Na_2_O) at 2–10%, calcium oxide (CaO) at 2–8%, as well as trace amounts of titanium dioxide and additional oxides, collectively reaching up to 28% [55]. Researchers have been actively exploring ways to employ red mud into both OPC manufacturing and alkali-activated binder formulations [84]. Incorporating a small percentage of red mud as a partial substitution for cement in GPC has been demonstrated to significantly enhance key mechanical properties, including Young’s modulus (E), compressive strength (CS), and failure strain in the resulting material [71].

Table 1 displays the chemical composition of frequently employed precursors in GP manufacturing. Notably, there is a noticeable variation in the chemical composition across different precursor types.

### 3.2. Activator

In GPC, alkaline activators, including liquid and solid, are generally employed to polymerize aluminosilicates. Alkaline activators (Aas) are used to initiate the polymerization of aluminosilicates to produce geopolymer GPC. The alkaline solutions utilized are often powerful and can contain compounds such as potassium hydroxide (KOH), sodium hydroxide (NaOH), potassium silicate (K_2_SiO_3_), sodium silicate (Na_2_SiO_3_), or a mix of these silicates and hydroxides. These activators dissolve the silicon (Si) and aluminum (Al) atoms, allowing them to recombine into the geopolymeric network [54,94]. The type of activators can impact the geopolymer material’s microstructure and mechanical properties, including durability, setting time, and strength [95]. Prior research has indicated that sodium-based alkali activators tend to exhibit greater activation efficiency in comparison to potassium-based activators for F fly ash [96]. Nevertheless, another study [97] discovered that the incorporation of potassium compounds in geopolymer systems resulted in heightened alkalinity compared to the utilization of NaOH. The majority of geopolymers are activated through the utilization of alkali activators; however, others are activated using acidic activators [98]. Acid-based activators offer a compelling alternative to the more commonly favored alkaline option. These activators are typically derived from either phosphate-based acids or humic-based acids [99]. Humic acids, as natural organic acids, are not commonly employed in this field of study due to the complex nature of their composition. Consequently, the application of acidic activation has predominantly centered on phosphate-based activators [99]. The most popular phosphate-based activator is phosphoric acid (PA). Furthermore, another type of phosphate-based activator in the field of geopolymers production is aluminum phosphate based activators, like Al(H_2_PO_4_)_3_, AlH_3_(PO_4_)_2_•3H_2_O, and Al(HPO_4_)_3_ [100].

## 4. Properties of Geopolymer Materials

This section is poised to delve into critical characteristics that define the performance and applicability of geopolymer materials. It extensively covers three pivotal aspects: compressive strength, durability, and the curing methods specific to geopolymer materials. These properties collectively contribute to the material’s suitability for a wide array of construction applications. Understanding and optimizing these attributes is paramount in ensuring that geopolymer concrete meets the rigorous engineering standards demanded by diverse projects.

### 4.1. Compressive Strength (CS)

The investigation of geopolymer mix properties revealed that various elements serve a crucial function in determining the compressive strength of these materials. Among these, Molar Ratios emerge as significant influencers [101]. The ultimate strength of alkali activated materials (AAM) is determined by the mixing design, including the ratios of silicon (Si) to aluminum (Al) [102], aluminum to sodium (Al/Na) [103], as well as the proportion of water to sodium (water/Na). Additionally, the reactivity of individual components in the mix proves to be an essential factor affecting the final strength of the AAM. Moreover, the curing temperature and time have a substantial impact. These parameters significantly affect the development of compressive strength in geopolymers [104]. The type of alkaline activator utilized also has a considerable effect on the material’s characteristics. The choice of activator may contribute to varying outcomes in terms of strength and overall performance [105]. Furthermore, the water content within the mix emerges as another critical determinant. The amount of water incorporated can significantly influence the CS of the resulting geopolymer [105]. Finally, the presence of calcium and other impurities was found to be a noteworthy factor. These impurities can introduce variability in the material’s properties and influence its ultimate strength [43]. Furthermore, Huseien et al. [106] performed a study on GP mortars, substituting granulated blast furnace slag (GBFS) with metakaolin (MK) at different proportions differing from 0% up to 15%. It was concluded that the CS of the resulting GP mortar after 28 days of curing exhibited an enhancement, rising from 42 MPa up to 63.1 MPa as the concentration of metakaolin increased, particularly within the range from 10% to 15%. Another research was performed by Abdullah et al. [107] to explore the effect of altering the dosage of alkaline activator and varying the proportion of sodium silicate (Na_2_SiO_3_) to sodium hydroxide (NaOH) in geopolymers derived from FA. Although the study did not reveal distinct patterns, it achieved a peak CS of 70 MPa. This was achieved by maintaining a ratio of 2 for FA/alkaline activator along with a ratio of 2.5 for Na_2_SiO_3_/NaOH. In the investigation led by Pavithra [108], the focus was on examining the impact of altering the sodium silicate/NaOH ratio in geopolymers derived from FA. It was found by the study that the optimal ratio of sodium silicate/NaOH was determined to be 1.5, resulting in a CS of 46 MPa. However, higher ratios contributed to a decline in the compressive strength. The decline was due to elevated sodium silicate levels, which raised the ratio of Si/Al, consequently causing a reduction in CS. In another investigation carried out by [109], the authors examined the behavior of FA-based geopolymers under different water content conditions, subjecting them to both heat and ambient temperature curing processes. The findings demonstrated a consistent trend where geopolymeric blends with lower water contents exhibited more condensed structures and experienced greater advancements in compressive strength, regardless of the curing method. Additionally, observations indicated that lower initial water content led to a rapid rate of CS enhancement in geopolymers cured at ambient temperature. However, in the case of heat-cured geopolymers, lower initial water content did not influence the gain rate of compressive strength. Tian et al. [110] noted that by substituting 20% of NaOH with CaO as the alkaline activator in alkali-activated copper tailings-based pastes, the CS increased from 35.6 up to 40.1 MPa. However, as the CaO substitution was further raised from 40% to 80%, there was a sharp decline in CS, dropping from 30 to 2 MPa. Additionally, it was found that the long-term CS of the pastes weakened with higher levels of CaO substitution. The research conducted by Duxson et al. [48] focused on the microstructure and composition of MK based geopolymers. It was observed that by utilizing sodium silicate (Na_2_SiO_3_) as an alkaline solution with a ratio of 1 for Al_2_O_3_/Na_2_O and a specific water-to-sodium oxide ratio of H_2_O/Na_2_O = 11, the CS of geopolymers experienced a substantial increase of approximately 400% as the ratio of Si/Al progressed from 1.15 to 1.90. However, the compressive strength began to decline at the highest Si/Al ratio of 2.15. Table 2 provides a summary of the research for different geopolymer concrete mixes, including the compressive strength (CS), curing temperature (CT), curing days (CD), molarity (M), and number of days for testing (D).

### 4.2. Geopolymer Materials Curing 

The curing stage of freshly prepared geopolymer concrete (GPC) holds great significance within the geopolymerization process, primarily due to its vital function in optimizing concrete quality [52]. This curing process also exerts a favorable impact on the final properties of GPC [142]. Typically, GPC is subjected to elevated-temperature curing through three distinct methods: steam curing, ambient curing, and oven curing regimes [52]. Ambient curing describes the method of curing geopolymers at room temperature. The specimens are cast and allowed to rest for a single day at 20 ± 3 °C [143,144]. Geopolymers cured under ambient conditions express an improvement in CS from 7 to 28 days [144]. Although ambient curing is considered the most cost-effective approach for curing geopolymers, it is essential to note that this method also entails the longest curing period [145]. On the other hand, steam curing is a specific curing procedure for geopolymers that is used to accelerate their development and improve their mechanical characteristics. The cast specimens are subjected to a controlled environment of higher temperature and high humidity, which is commonly accomplished by exposing them to steam [146]. Oven curing is the process of curing GPC, which requires heating the specimens in an oven. This approach has demonstrated its efficacy in enhancing the performance of the GPC, leading to heightened compressive strength compared to alternative curing procedures [147]. The temperature parameters for oven curing typically range between 40 °C and 120 °C [52]. The selection of the precise temperature within this range relies on the targeted strength and the characteristics of the GPC undergoing the curing process [145].

Numerous studies have been performed to investigate the impact of several curing conditions on the properties of geopolymer pastes. The reported curing temperatures spanned between 40 °C and 85 °C to finish the geopolymerization reaction by [97]. The curing process of alkali-activated FA was assessed by [148], utilizing activator-to-FA ratios of 0.25 and 0.30 at temperatures of 65 °C and 85 °C. It was found that geopolymers’ CS go through significant enhancement when cured at 85 °C for 24 h in comparison to those cured at 65 °C. However, the increment in strength was notably less noticeable when the curing duration extended beyond 24 h. Additionally, curing metakaolin-based geopolymer concrete in a controlled relative humidity (RH) oven has no advantages over ambient curing followed by mild heating (40–60 °C) in sealed containers [127]. In another study, Heah et al. [149] performed experiments on kaolin-based GPC under heat and ambient curing conditions. Observations showed that due to the low initial strength, the curing at ambient conditions was considered as not feasible. Another work by Yunsheng et al. [150] examined the impact of curing conditions on the strength of slag-based GPC. The study revealed that ambient temperature curing resulted in weaker concrete compared to steam curing. Steam curing at 80 °C for 2 h led to a 19.14% strength increase over 3-day ambient curing. Extending steam curing to 4 and 8 h boosted the strengths by 46.03% and 53.16%, respectively. Notably, the study demonstrated a remarkable peak compressive strength of 70 MPa through 2 h autoclave curing, emphasizing the efficacy of customized curing methods for optimizing slag-based geopolymer concrete strength.

**Figure 4:**
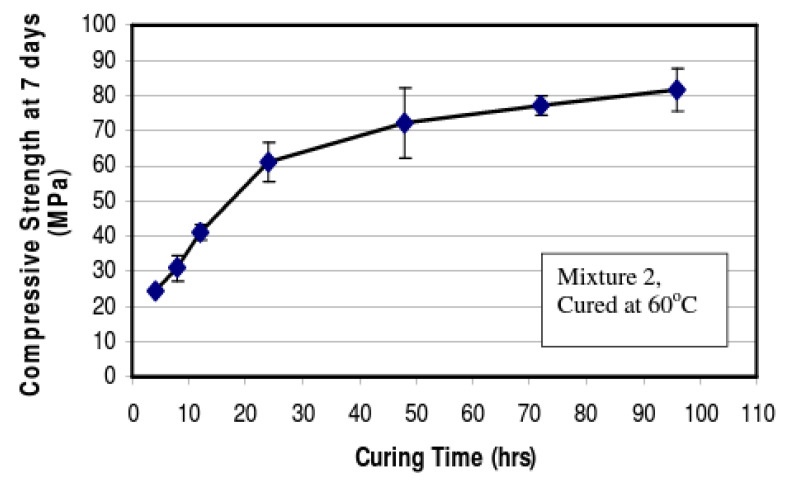
Impact of curing duration on the compressive strength. Reproduced with permission from [151].

The development of compressive strength in geopolymer concrete is significantly affected by the duration and temperature of the curing process. This relationship is depicted in Figure 4 below and within the study by Hardjito and Rangan [151]. In their experiment, cylindrical specimens measuring 100 by 200 mm were subjected to heat curing at 60 °C. The duration of this curing ranged from 4 to 96 h. It was observed that an extended curing period enhanced the polymerization reaction, which, in turn, increased the compressive strength of the material. The strength grew swiftly within the first 24 h of curing, after which the progression in strength continued at a slower pace.

### 4.3. Durability of Geopolymer Materials

**Figure 5:**
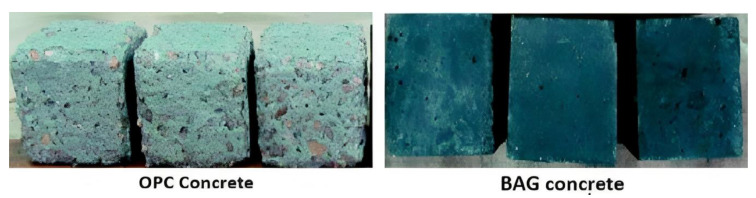
Visual condition of concrete samples after an 18-month exposure to sulfuric acid. Reproduced with permission from [158].

**Figure 6:**
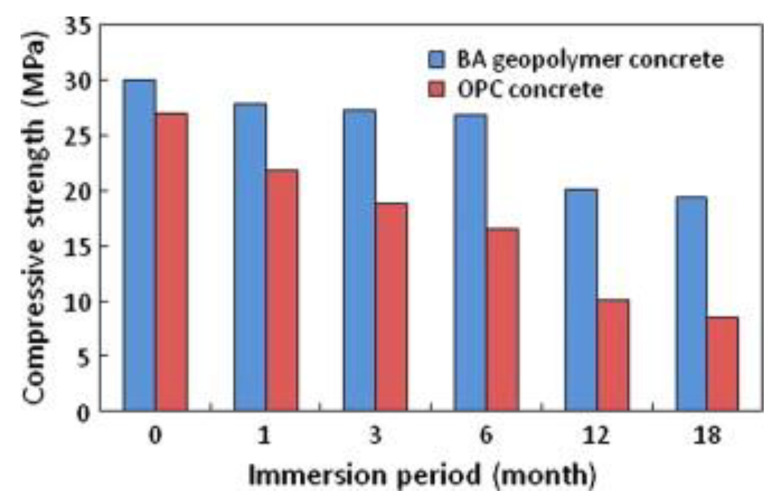
Compressive strength of concrete samples following 18 months of sulfuric acid exposure. Reproduced with permission from [158].

Durability refers to the material’s ability to withstand different environmental conditions, various forms of chemical attack (such as carbonation, sulfate, chloride, and acid attacks), physical attacks (including freeze-thaw cycles and elevated temperatures), mechanical attacks (like abrasion, erosion, and cavitation), as well as potential construction-related issues (such as inadequate consolidation and curing). This quality ensures that the material maintains its performance throughout its service life [104]. In a study performed by Pasupathy et al. [152], a comprehensive examination was undertaken to assess the carbonation resistance of geopolymeric concrete derived from a combination of slag and fly ash over an eight-year period of exposure to the environment. The findings showed that the carbonation rate of GPC was remarkably affected by the specific mix design of its materials. Notably, the mixture categorized as Type 1 GPC, consisting of 75% FA, 25% GBFS, and an extra Na_2_SiO_3_ activator, exhibited lower carbonation resistance in comparison to OPC concrete. On the other hand, Type 2 geopolymer, comprising 70% FA, 30% GBFS, and lacking the additional Na_2_SiO_3_ activator, displayed a carbonation resistance similar to that of OPC concrete. In another study, Elyamany et al. [153] performed a study to analyze the factors affecting the resistance of geopolymer mortar to magnesium sulfate MgSO_4_ where various geopolymer mortars were compared with OPC mortar by immersing them for up to 48 weeks in a solution with 10% magnesium sulfate. The findings revealed that higher cure temperatures, greater molarity of sodium hydroxide solution, and a decrease in the proportion of alkaline solution to binder improved the geopolymer mortar’s resistance to magnesium sulfate MgSO_4_. As a result, geopolymer mortars demonstrated improved durability in MgSO_4_ solution in comparison to OPC mortars. Yang et al. [154] examined the effects of exposing FA geopolymer pastes to a 3% NaCl solution for 72 h. The findings were positive, particularly when slag was added to the FA-based geopolymer. This inclusion of slag served to fill the pores within the material, reducing its vulnerability to the entry of chloride ions. Moreover, the study conducted by Sun and Wu [155] illustrated that F-type fly ash, characterized by its low calcium content, exhibited good resistance to freeze-thaw cycles when compared to Portland cement. Furthermore, Bakharev [156] examined the durability of geopolymer materials created using alkaline-activated FA and subjected them to 5% solutions of acetic and sulfuric acids. The geopolymer materials demonstrated notably better performance in comparison to OPC pastes. Moreover, Marvila et al. [157] investigated the behavior of MK-based geopolymers activated using sodium hydroxide and silicate when subjected to elevated temperature environments. It was confirmed that the mechanical characteristics of the materials in question remained intact even after exposure to temperatures as high as 1050 ºC. Ariffin and colleagues [158] conducted a long-term study in which geopolymer concrete (GPC), composed of a mixture of pulverized fuel ash and palm oil fuel ash, was subjected to a 2% sulfuric acid solution for a duration of 18 months. Observational assessments revealed that the blended ash geopolymer (BAG) concrete samples exhibited no notable alteration in their external appearance, whereas ordinary Portland cement (OPC) concrete samples displayed pronounced degradation, as illustrated in Figure 5. The BAG samples experienced an 8% loss in weight, which was considerably less than the 20% weight reduction seen in the OPC concrete samples. Regarding structural integrity, BAG concrete showed a decrease in strength by 35% over the 18-month period, whereas OPC concrete suffered a 68% reduction in strength within just 30 days, with further significant deterioration noted at the 18-month point, as depicted in Figure 6.

## 5. Applications of Geopolymers

**Figure 7:**
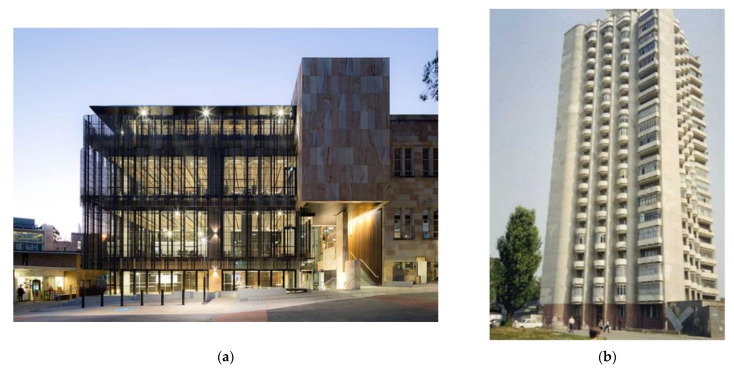
(**a**) The University of Queensland’s Global Change Institute [166]; (**b**) building constructed in 1994 with AAS concrete, completed in Lipetsk, Russia [162].

Concrete consumption in the construction sector has grown recently, and the demand for concrete is expected to grow more and more in the future [71]. Geopolymer concrete can be among the finest substitutes for ordinary concrete due to its advantageous characteristics. Even though geopolymer concrete has not yet gained widespread acceptance, its use or the use of its derivatives is expanding quickly on a global scale [159]. The first 20-story residential building consisting of alkali-activated concrete (AAC) without any Portland cement was constructed in Lipetsk, Russian Federation, in 1989 [160]. In 2013, the University of Queensland’s Global Change Institute utilized precast geopolymer concrete beams to create a multi-story building, marking the first time that geopolymer concrete has actually been employed in construction [161]. Numerous researchers have shown that geopolymer may be used as a highway infrastructure repair material [162]. Furthermore, geopolymer precast panels cured under ambient conditions were used as a retaining wall for a private residence in Toowoomba, Australia [161]. The most significant benefit of geopolymer concrete over regular concrete is its good durability, making it ideal for usage in places such as offshore structures, tanks, and any concrete elements subjected to corrosive environments such as sulfate or chloride attack [163]. Fly ash-based geopolymer concrete demonstrated impressive fire resistance compared to Portland cement concrete due to the inclusion of industrial pozzolanic waste [71]. The main potential applications for geopolymers are road construction and maintenance, tanks, boat ramps, offshore structures, retaining walls, precast members, bridge structures, and other structural members [54,159,162]. Other possible uses for geopolymers include geopolymer coating, the solidification/stabilization of hazardous wastes, stabilizing landfills, the construction of landfill baseliners with low permeability, thermal insulation, water control structures, and sustainable repair materials [159,162,164,165]. Figure 7 provides visual references to two different constructions that used geopolymer instead of Portland cement.

## 6. The Environmental Impacts of Geopolymers

**Figure 8:**
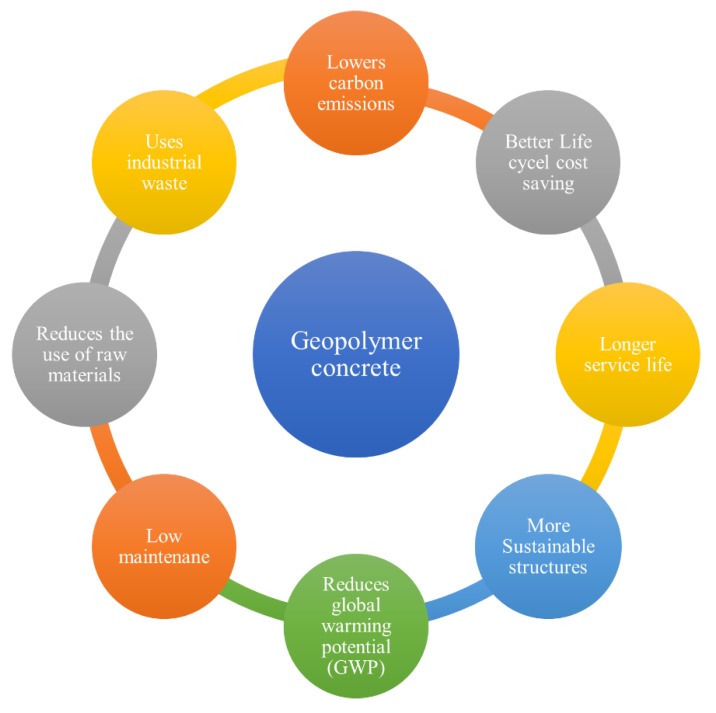
The advantages of using geopolymer instead of Portland cement.

The construction industry is an important sector that plays a part in the national economy and the prosperity of nations [30]. Thus, the construction sector is in charge of producing huge amount of waste and emitting substantial amounts of greenhouse gases (GHGs) into the environment [167]. Portland cement is the main material utilized in the construction of numerous infrastructure projects worldwide. The manufacturing of cement may pollute the environment’s water and air [168]. Cement production emits a significant quantity of pollutants such as CO, CO_2_, SO_2_, NOx, and particulates due to the use of fossil fuels and the decomposition of limestone [169]. It also requires a substantial quantity of energy and emits a massive amount of CO_2_ into the environment (5–7% of the global CO_2_ emissions) [170]. The enormous amount of natural resources required to produce cement has also led to the over-exploitation of natural resource reserves, with a concomitant degradation of environmental aesthetics and alteration of ecosystem structures [162]. As a result, cement substitutes are constantly being researched and developed in order to enhance the construction industry’s sustainability. Among these substitutes, geopolymers have attracted a lot of scientific attention. Geopolymer concrete is regarded as the updated generation of concrete since it is environmentally beneficial and eliminates the need for regular OPC in the manufacturing of concrete [171]. It was stated that GP composites produced from industrial wastes, including slags, FA, and other aluminosilicate materials, might participate in decreasing carbon emissions by 80%, making geopolymers a greener substitute to cement [172]. The scale of the reduction is affected by a variety of factors, including raw material transportation, methods of production, and the type of materials utilized [173]. An LCA was conducted to compare the global warming potential (GWP) of regular Portland cement concrete to geopolymer concrete. It was found from the results that GPC experienced a reduction in GWP of 26–45% compared to regular Portland cement concrete [173,174]. Further evidence of the geopolymer’s environmental friendliness comes from the usage of fly ash, which will end up in landfills if it is not used in the production of geopolymers [175]. However, geopolymer concrete does not compare favorably to Portland cement concrete when additional ecological impact considerations are taken into account. This is mostly attributed to the production of sodium silicate (Na_2_SiO_3_) and sodium hydroxide (NaOH) [19,174]. Bajpai et al. [86] evaluated fly ash-based geopolymer’s environmental impact, and one of the main key findings of the study was that utilizing an alkaline activator had the highest negative impacts. Geopolymer as a technology prevents potentially toxic components from leaching into the environment, diverts waste streams from landfills, and enables the replacement of carbon-intensive materials like cement, complying with the circular economy paradigm while having the potential to achieve high material efficiency performance [176]. Thus, using geopolymers as a sustainable substitute for OPC materials would lead to a considerable decrease in GHG emissions and raw material use and provide a route to efficient waste management [177]. The benefits of using geopolymer concrete instead of Portland cement concrete in multiple construction applications are illustrated in Figure 8.

The life-cycle assessment (LCA) is a commonly used approach for examining the environmental effects of products or processes across their life span, from raw materials extraction to waste disposal [30,31,178,179,180]. Multiple studies have been conducted to evaluate the environmental performance of geopolymer using LCA. One study published in the *Journal of Cleaner Production* by Habert et al. [34] utilized the LCA approach to conduct a thorough environmental assessment of the GPC manufacturing process. The findings suggested that the manufacturing of several types of GPC has a reduced impact on global warming potential (GWP) compared to ordinary Portland cement concrete (OPCC). Nevertheless, the study also indicated that the GP concrete’s manufacturing process has the worse environmental impact compared to OPCC’s other impact categories. Similar results were found by Graces et al. [181] when they indicated that self-healing GPC outperformed OPCC in terms of GWP but performed worse in terms of other environmental impact categories. In another study, Imtiaz et al. [182] conducted a comparative LCA and found that substituting GPC for OPCC resulted in reduced impact in categories like acidification potential (AP), photochemical oxidant formation (POCD), and climate change (GWP). However, using GPC led to an increase in categories, such as marine aquatic ecotoxicity (MAE), human toxicity (HT), freshwater aquatic ecotoxicity (FWAE), terrestrial ecotoxicity (TE), eutrophication potential (EP), and ozone depletion potential (ODP) due to the existence of alkaline activators like sodium hydroxide NaOH and sodium silicate Na_2_SiO_3_ in the GPC. Furthermore, Salas et al. [19] concluded in their LCA study that when sodium hydroxide was manufactured with solar salt, GPC exhibited advantageous performance over OPC in categories like GWP, abiotic depletion for fossil fuels (ADPF) and EP, while GPC performed worse in AP, POCP, and ODP. Additionally, Garces et al. [183] presented in their study that self-healing geopolymer concrete (SHGPC) had a lower GWP than OPC and had a higher impact in the other environmental impact categories. In another study, Fernando et al. [184] utilized LCA to evaluate the environmental performance of fly ash geopolymer (FA-GPC) and blended fly ash rice husk ash (FA-RHA-GPC) alkali activated concrete. The findings showed that the alkali activator was responsible for the high environmental impact of both FA-GPC and FA-RHA-GPC when compared to OPCC. In addition, an LCA was performed by Asadollahfardi et al. [185] comparing the environmental performance of GPC to OPCC, and it was presented by the study that OPCC has the lowest environmental impact in all categories in the manufacturing process, except GWP, which had a higher value in OPCC in comparison to GPC, resulting from the consumption of cement. Overall, these LCA studies suggest that GPC has a reduced environmental impact compared to OPCC; however, the environmental performance is related to the type of the activator and the source of the raw materials. Table 3 represents the results for some LCA studies found in the literature.

### Global Warming Potential of Geopolymers

**Figure 9:**
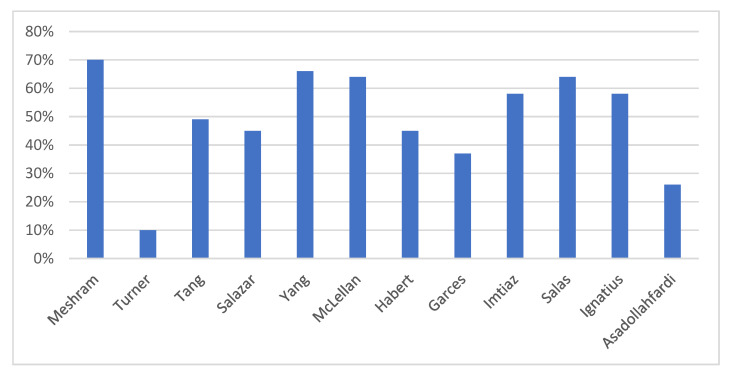
Maximum reduction ratio in GWP for GPC compared to OPCC.

The global warming potential (GWP) assesses the increase in average global temperature over a period of time caused by various GHGs such as N_2_O, CO_2_, CH_4_, SF_6_, chloro-fluoro-carbons (CFC), and hydro-chloro-fluorocarbon (HFC) [186]. The GWP caused by geopolymer concrete (GPC) is a popular trend nowadays since it demonstrates that GPC has a lower potential to contribute to global warming than ordinary Portland cement concrete (OPCC). The Intergovernmental Panel on Climate Change (IPCC) has stated that replacing the current concrete binders with geopolymers or binders with high FA and slag content was found to be a viable alternative for minimizing carbon dioxide (CO_2_) emissions [187]. Numerous studies have compared geopolymer to ordinary Portland cement and found a reduction in GWP, as shown in Figure 9. 

In a study conducted by Meshram and Kumar [186], it was found that FA and slag-based geopolymer cement (GC) reduce the GWP by 70% in comparison to OPC, while GWP was reduced by 61% when FA and cement-based GC were used. GWP through LCA was also evaluated by [36] of GPC based on 70% natural volcanic pozzolan and 30% granulated blast furnace slag (GBFS). The findings showed less carbon footprint by approximately 45% when GPC was used instead of OPCC. Additionally, the carbon dioxide-equivalent (CO_2-e_) emissions were compared by [37] between GPC and OPCC, and it was concluded that the CO_2_ footprint of GPC was approximately 10% less than its comparable OPCC. Habert [34] found a 44.92% improvement in GWP when GPC was used instead of OPCC. Salas [19] compared three different scenarios for producing GPC; in scenarios S1 and S2, sodium hydroxide NaOH was produced locally in Ecuador using local raw materials, whereas, in scenario S3, sodium hydroxide was obtained from Europe. It was observed by [19] that using environmentally friendly NaOH derived from nearby solar salt may reduce the GWP by up to 64% compared to conventional concrete. Another study by [181] showed that GPC achieved a 37% reduction in GWP compared to OPCC, while only an 8% reduction was spotted when self-healing geopolymer concrete SHGPC was used. Similarly, Imtiaz [182] compared OPCC to GPC and recycled aggregate-based geopolymer concrete (RAGC) through LCA. The findings showed that the utilization of GPC instead of OPCC resulted in a reduction in GWP by 57.3%; however, the addition of recycled aggregates in the geopolymer mix gave an extra reduction in the total impact of GWP. In a study conducted by Tang [188], two types of geopolymers were analyzed, one made of fly ash (G-FA), while the other was made from cenospheres (G-C),;these were compared to OPC made with natural aggregates (NAC). The findings indicated that producing G-FA and G-C revealed lower GWP by 32%, and 49%, respectively, in comparison to NAC. Furthermore, Yang et al. [177] found that compared to OPC, alkali-activated (AA) concrete exhibited a notable decrease in CO_2_ emissions, ranging between 55% and 75%. However, the extent of this decrease might vary based on the amount and type of alkali activators employed in concrete production. McLellan et al. [35] noted that geopolymer cement made from local materials in Australia without the use of external heat led to a remarkable decrease of 44–64% in CO_2eq_ emissions released into the environment compared to OPC production. Additionally, Garces et al. [183] conducted an LCA of GPC, including microcapsules for self-healing. The study revealed that the self-healing geopolymer concrete (SHGPC) was better than OPCC in terms of GWP, with a 58% reduction in CO_2eq_ emissions. Moreover, Asadollahfardi et al. [185] indicated a 26% reduction in GWP when geopolymer concrete was compared to OPCC. However, when Fernando et al. [184] conducted an LCA for three types of concrete, namely fly ash (FA) geopolymer concrete, blended FA rice husk (RHA) alkali-activated concrete, and OPCC, it was found that the GWP had similar values for all mixes used in the study. Overall, the GWP of GPC is remarkably lower than traditional Portland cement concrete, leading to a promising sustainable alternative in the construction industry, as shown in Table 4.

## 7. Conclusions

In this study, an extensive examination of multiple research and applications related to GPC was undertaken. Based on this investigation, the following conclusions may be derived:

- Geopolymers derive their strength from abundant sources of active silicon and aluminum. The raw constituents of geopolymers typically encompass BFS, FA, MK, RHA, and others.
- GPC is an environmentally friendly building material with outstanding mechanical characteristics. It is seen as an attractive alternative for OPC concrete, which would be achievable if sufficient industrial and agricultural waste materials were available. Adopting geopolymer concrete instead of traditional OPC concrete could cause an 80% decrease in carbon dioxide emissions related to concrete manufacturing.
- It was observed that the ultimate properties of the geopolymer are contingent on its chemical composition, with the elements Al, Na, H_2_O, and Si being pivotal in the formation of the dominant N-A-S-H gel and, consequently, influencing the chemical attributes of the geopolymer.
- The findings from this study suggest that geopolymer concrete shows substantial promise and feasibility as an eco-friendly construction material. It holds potential as a possible substitute for conventional concrete in future applications.
- The study found that curing temperature, silicate content, and the alkaline solution-to-binder proportion are the primary factors significantly impacting the compressive strength of geopolymer concrete.
- The utilization of an alkaline activator plays a significant role in environmental impact, particularly in the case of GPC. Therefore, it is crucial to carefully choose the suitable source of alkaline activators for the GPC mixture.
- Employing waste materials in producing activated alkali substances offers economic advantages and significant environmental benefits by reducing reliance on Portland cement. Additionally, this approach addresses the challenges linked with the disposal of substantial quantities of waste, including ash from coal-fired thermoelectric plants and slag from metal production, mitigating potential environmental hazards.
- The reactivity of geopolymers is notably affected by both curing duration and temperature. Furthermore, factors such as particle size and water content play crucial roles in altering the durability of these materials.
- Geopolymers are not strictly an alternative aiming to rival the established Ordinary Portland Cement (OPC) industry on a worldwide scale. Instead, they can be seen as a technological advancement that cement manufacturers can adopt to diversify their portfolio of cement-based products for the market.

## 8. Future Directions

- Only a limited number of researchers have undertaken experiments concerning the structural applications of GPC. Therefore, there is a pressing need for more extensive research in this field to facilitate the widespread adoption of GPC applications within the construction industry. The utilization of GPC appears to hold significant promise in advancing sustainable construction practices in this sector.
- Analyzing the environmental and economic implications of GPC usage is crucial. Conducting a thorough evaluation of its impacts, both in terms of costs and sustainability, can raise awareness and promote its wider adoption. Furthermore, such research attempts can provide valuable insights into innovative approaches for further mitigating the environmental footprint and expenses associated with GPC.
- One of the main challenges confronting the widespread acceptance of geopolymerization is the entrenched dominance of OPC within the industry. Additionally, the industry tends to be cautious and conservative when it comes to adopting new technologies and products that could potentially replace established ones. Overcoming these obstacles will necessitate sustained and intensified efforts from the research community.
- Despite the multitude of field applications in the construction industry, there is a pressing requirement for a practical code of practice specifically tailored for geopolymers. The formulation of these materials should be grounded in extensive research and field data to facilitate widespread adoption by consumers.
- The rheological characteristics of alkali-activated specimens derived from different source materials remain unexplored territory and necessitate further investigation.
- Whereas GPC has been in existence for some time, there remains a necessity for conducting more extensive, long-term studies. Unlike OPC concrete, there is a restricted comprehension of GPC’s durability, particularly concerning formulations using unconventional precursors. Therefore, alongside short-term investigations, there should be an increased emphasis on studying its long-term performance. Employing various accelerated testing methods could prove beneficial in thoroughly evaluating the extended performance of GPC.

## Figures and Tables

**Table 1:** The chemical composition of RHA, BFS, RD, MK, FA, and SF (wt, %).

Precursors	Reference	Composition (%)										
		SiO_2_	Al_2_O_3_	Fe_2_O_3_	CaO	MgO	MnO	K_2_O	Na_2_O	P_2_O_5_	TiO_2_	SO_3_
FA	[85]	65.9	24	2.87	1.59	0.42	0.06	1.44	0.49	0.19	0.92	-
FA	[86]	52.83	21.50	10.49	6.44	0.89	-	1.76	0.82	1.75	1.6	-
FA	[87]	62.04	25.50	4.28	3.96	1.27	-	-	0.46	0.31	1.33	0.73
FA	[88]	61.86	-	-	-	0.86	-	-	-	-	-	0.28
FA	[72]	50.70	28.80	8.80	2.38	1.39	-	2.40	0.84	-	-	0.30
BFS	[85]	36	13.8	0.3	42.6	5.8	0.4	0.27	0.21	0.10	0.8	0.56
BFS	[69]	35.80	13.21	1.97	35.68	9.76	-	0.57	0.48	-	-	0.21
BFS	[89]	32.5	13.7	0.8	45.8	3.3	0.4	0.5	0.3	0	0.7	1.8
BFS	[87]	34.11	15.36	0.83	35.99	6.58	1.07	0.62	0.4	-	2.41	2.50
BFS	[88]	32.9	-	0.7	41.3	5.9	-	-	0.45	-	-	0.21
RHA	[69]	89.47	0.83	0.53	0.68	0.37	-	0.17	0.22	-	-	0.12
RHA	[89]	93.46	0.58	0.52	1.03	0.51	-	1.82	0.08	1.6	0	0.6
RHA	[90]	89.17	0	0.41	0.61	1.22	-	1.12	7.29	-	0.03	-
RM	[91]	16.51	28.05	30.32	2.22	0.7	0.11	0.26	8.70	-	4.29	-
RM	[92]	27.544	30.591	4.603	25.478	0.818	0.012	3.82	-	-	5.151	1.422
MK	[93]	54.4	39.4	1.8	0.1	-	0.01	1.0	-	0.1	1.6	-
MK	[75]	50.995	42.631	2.114	1.287	0.127	0.006	0.337	0.284	0.051	1.713	0.439
MK	[89]	51.7	40.6	0.64	0.71	0.96	0.08	2	0.31	0.2	3	0.1
SF	[86]	92.39	1.41	0.154	0.547	-	-	<1	-	2.32	<1	-
SF	[88]	92.98	-	1.49	0.32	0.57	-	0.51	0.47	-	-	0.57
SF	[72]	93.67	0.83	1.30	0.31	0.84	0.84	1.10	0.40	-	-	0.16

**Table 2:** Summary of research for different geopolymer concrete mixes.

Reference	Source	Si/Al	M	CT (°C)	CD (h)	D (Day)	CS (MPa)
[111]	FA + OPC	2.5	10	80	24	28–365	44–55
[112]	FA	1.5–3.9	15	80	24	3–365	22.5–60.7
[113]	Nano silica + FA	2.29–4.10	8	80	24	28	37.2–47.3
[114]	FA	2.89	12	70–800	24	28	11.93–17
[115]	Metakaolin	2–6	6–7	20	-	28	5.4–34.9
[116]	Metakaolin	2.25–4	10	Ambient, 50 and 75	24	3–90	2–66
[117]	Metakaolin	3.5–3.8	12	85	2	-	2–48
[118]	FA	2.3	16	Ambient and 60	24	3–28	8–50
[119]	FA	7.7	8–16	Ambient (23)	-	28	7.6–21.5
[120]	FA + GBFS	-	10–12	Ambient (20)	-	1–56	2–60.1
[121]	Natural pozzolan+ nano-silica	2.47–4.17	14	60	168	1–28	7.32–44.97
[122]	FA	1–1.88	-	75	16	-	33.45–41.02
[123]	FA	2.1	8–14	60–90	24–48	3–7	20–49
[11]	Metakaolin	1–5	-	60	6	7	2.1–36.8
[124]	FA + RHA	2	-	Ambient	-	7–90	17.2–48.7
[125]	FA	1.6	14	25	24	7–70	7.1–48.2
[125]	Bottom Ash	2.16	14	25	24	7–70	0.2–1.1
[125]	FA + BA	1.6–2.16	14	25	24	7–70	0.8–12.7
[126]	Copper tailings + FA	1.89–7.78	5–15	60	-	2–28	1.37–21.2
[127]	Metakaolin	1.86–2.11	7.2	Ambient then 40–60	24 + 24	7	57–61
[128]	FA	1.5–5.1	12–16	Ambient then 70	24 + 24	7	16–64
[129]	RM + FA	1.5–2.75	6–12	60	24	7	5.3–38
[130]	FA + Alccofine	-	16	Ambient to 90	24	3–28	2.5–73
[131]	FA + GBFS	-	12	75	18	28	51.1–53.2
[132]	Metakaolin	1–3	11–18	75	24	7	0.4–64
[133]	FA + GBFS	1.8	8	Ambient	-	7–28	12.88–45.55
[134]	FA	2.1	8–16	24–120	6–72	3–28	13–56
[135]	Gold mine tailing	1–11	10	60–110	-	5	1.23–18.10
[136]	FA	-	3–9	50	72	3–7	45–81
[137]	FA + RHA	2.1	8	Hot gunny then Ambient	24	3–56	3.19–50.96
[138]	Palm oil-fuel ash (POFA) + FA + oil-palm shell (OPS)	3.43–6.17	14	65	48	3- 28	7.3–30.1
[139]	GGBFS + MK + POFA	-	14	65	24	3–28	24.7–41.5
[140]	FA + OPC	1.765–2.018	14	20–23	-	3–90	4–46
[141]	FA	2.6–2.9	12	80	24	7	28.99–46.18

**Table 3:** Life-cycle assessment results from different studies.

Reference	Type of Concrete	ADPF	GWP	ODP	HT	FWAE	MAE	TE	POCP	AP	EP
[34]	GPC	1.19	168.5	1.39 × 10^−5^	105.4	27.01	0.000459	1.77	3.65 × 10^−2^	0.82	0.0796
	OPCC	0.61	305.9	8.74 × 10^−6^	18.9	2.52	0.00968	0.45	1.67 × 10^−2^	0.45	0.0683
[181]	OPCC	757.42	454.5937	4.66 × 10^−9^	-	-	-	-	4.49 × 10^−2^	0.8217	0.1647
	GPC	2933.205	285.0813	2.24 × 10^−8^	-	-	-	-	9.30 × 10^−2^	1.6067	0.1337
	SHGPC	6428.252	417.1633	2.66 × 10^−6^	-	-	-	-	1.65 × 10^−1^	2.2264	0.2226
[182]	OPCC	-	264.181	0	0.8952	1.78 × 10^−7^	4.58 × 10^−5^	6.32 × 10^−31^	9.63 × 10^−2^	1.01904	0.07922
	RAC	-	261.315	0	0.886	1.68 × 10^−7^	4.48 × 10^−5^	6.3 × 10^−31^	4.11 × 10^−2^	1.01165	0.0788
	GPC	-	112.743	5.59 × 10^−5^	33.7	40.94	136.45	0.0107	7.77 × 10^−2^	0.60119	0.11483
	RAGC	-	111.377	5.59 × 10^−5^	33.68249	40.94	136.45	0.0107	5.13 × 10^−2^	0.59769	0.11463
[19]	OPC	1213	302	0.13	-	-	-	-	2.60 × 10^−2^	0.674	0.174
	GPC-S1	900	110	1.61	-	-	-	-	2.90 × 10^−2^	0.727	0.155
	GPC-S2	1480	163	1.67	-	-	-	-	4.90 × 10^−2^	1.237	0.201
	GPC-S3	2796	254	1.78	-	-	-	-	5.60 × 10^−2^	1.263	1.245
[183]	OPCC	1892	333.65	4.19 × 10^−6^	-	-	-	-	1.30 × 10^−2^	0.78	0.265
	GPC-1	2443	207.51	1.10 × 10^−5^	-	-	-	-	4.30 × 10^−2^	1.21	0.265
	GPC-2	1618	138.89	7.75 × 10^−6^	-	-	-	-	3.20 × 10^−2^	0.94	0.195
	GPC-3	2127	178.1	9.50 × 10^−6^	-	-	-	-	3.90 × 10^−2^	1.11	0.243
	SH1GPC-2	1817	171.076	8.73 × 10^−6^	-	-	-	-	3.60 × 10^−2^	1.09	0.2226
	SH1GPC-3	2339	212.26	1.05 × 10^−5^	-	-	-	-	4.20 × 10^−2^	1.27	0.273
	SH2GPC-2	4558	252.06	1.74 × 10^−5^	-	-	-	-	8.50 × 10^−2^	1.43	0.34
	SH2GPC-3	5247	298.19	1.98 × 10^−5^	-	-	-	-	9.50 × 10^−2^	1.63	0.397
[184]	100 PC	1.45 × 10^−2^	319	9.96 × 10^−6^	20.7	6.95 × 10^−1^	2.65 × 10^0^	-	1.89 × 10^−2^	0.65	0.251
	100% FA	2.32 × 10^0^	327	3.63 × 10^−5^	298.4	7.77 × 10^1^	1.13 × 10^−1^	-	2.66 × 10^−2^	0.758	0.209
	90%FA-10%RHA	2.31 × 10^0^	326	3.62 × 10^−5^	2.98 × 10^2^	7.77 × 10^1^	1.05 × 10^−1^	-	2.65 × 10^−2^	0.756	0.208
[185]	OPCC	-	386.44	-	35.68	-	-	-	-	0.84	0.159
	GPC	-	286.85	-	72.35	-	-	-	-	1.11	0.183

**Table 4:** The reduction ratio in GWP when geopolymer was used instead of ordinary Portland cement.

Reference	Typer of Concrete/Cement	GWP	Reduction in GWP	Reference	Type of Concrete/Cement	GWP	Reduction in GWP
[186]	OPC	895		[34]	OPCC	305.9	
	GC/FA + slag	267	70%		GPC	168.5	44.92%
	GC/FA + cement	351	61%	[181]	OPCC	454.5937	
[37]	OPCC	354			GPC	285.0813	37.30%
	GCC	320	10%		SHGPC	417.1633	8.23%
[188]	NAC	704		[182]	OPCC	264.181	
	G-C	360	49%		GPC	112.743	57%
	G-FA	477	32%		RAGC	111.377	58%
[36]	OPCC	381.17		[19]	OPCC	302	
	GPC	210.9	45%		GPC- S1	110	64%
[177]	OPCC	323			GPC- S2	163	46%
	AA GGBS	110	66%		GPC- S3	254	16%
	AA FA	160	50%	[183]	OPCC	333.65	
	AA MK	187	42%		GPC-1	207.51	38%
[35]	OPC	760			GPC-2	138.89	58%
	GP1	404	47%		GPC-3	178.1	47%
	GP2	271	64%		SH1GPC-2	171.076	49%
	GP3	310	59%		SH1GPC-3	212.26	36%
	GP4	425	44%		SH2GPC-2	252.06	24%
[184]	OPCC	319			SH2GPC-3	298.19	11%
	100% FA	327	−3%	[185]	OPCC	386.44	
	90%FA-10%RHA	326	−2%		GPC	286.85	26%

## Data Availability

All data is contained within the article.
